# Supplementary material for: Protein language models reveal evolutionary constraints on synonymous codon choice
Source: bioRxiv. 2025 Aug 5:2025.08.05.668603. Preprint. [Version 1] doi: 10.1101/2025.08.05.668603 (PMC12340807; doi:10.1101/2025.08.05.668603)
Supplement: Supplement 1 [file NIHPP2025.08.05.668603v1-supplement-1.pdf]

bioRxiv preprint doi: https://doi.org/10.1101/2025.08.05.668603; this version posted August 5, 2025. The copyright holder for this preprint (which was not certified by peer review) is the author/funder, who has granted bioRxiv a license to display the preprint in perpetuity. It is made available under aCC-BY 4.0 International license.

SUPPLEMENT

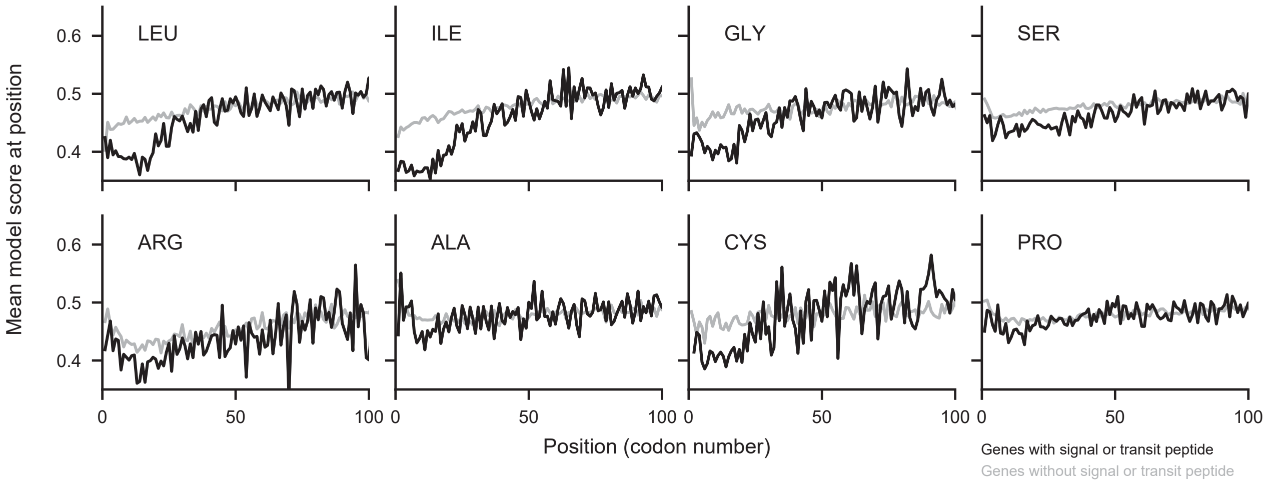

Figure S1: Signal and transit peptides are encoded with slower codons for all amino acids. The mean model score at each position with a given amino acid, averaged across all genes with a signal or transit peptide in black and across all genes without a signal or transit peptide in grey. For all amino acids, the first 50 codons are predicted to be significantly slower in genes with signal or transit peptides compared to other genes. The effect size is strongest for isoleucine (0.73) and leucine (0.64) and weakest for alanine (0.15) and proline (0.12).

bioRxiv preprint doi: https://doi.org/10.1101/2025.08.05.668603; this version posted August 5, 2025. The copyright holder for this preprint (which was not certified by peer review) is the author/funder, who has granted bioRxiv a license to display the preprint in perpetuity. It is made available under aCC-BY 4.0 International license.

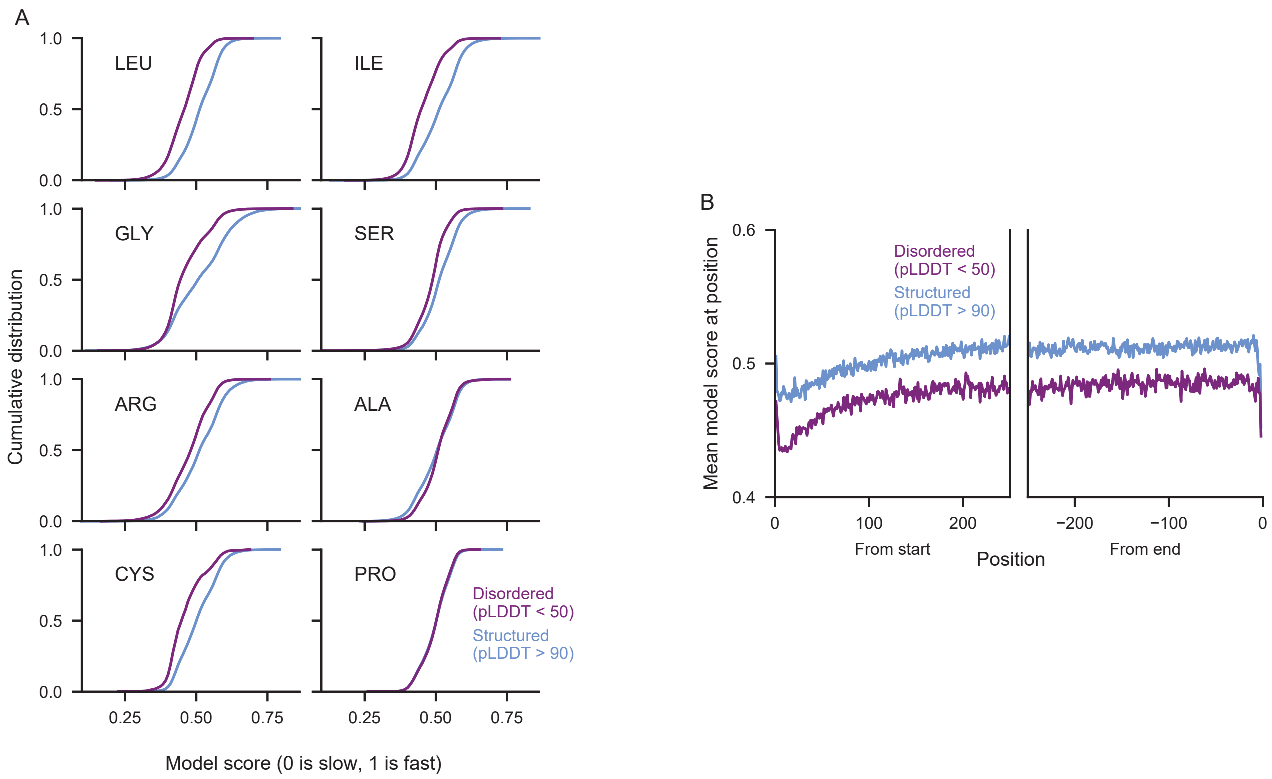

Figure S2: Structured regions predicted to be encoded with faster codons regardless of amino acid or position. A) The cumulative distribution of model scores for positions in structured (pLDDT > 90) and disordered (pLDDT < 50) regions, plotted individually for each amino acid. Positions in structured regions are predicted to have significantly more fast codons than positions in disordered regions for all amino acids except proline and alanine. B) The mean model score at positions in structured (blue) or disordered (purple) regions, plotted by position from the start and from the end of the gene.

bioRxiv preprint doi: https://doi.org/10.1101/2025.08.05.668603; this version posted August 5, 2025. The copyright holder for this preprint (which was not certified by peer review) is the author/funder, who has granted bioRxiv a license to display the preprint in perpetuity. It is made available under aCC-BY 4.0 International license.

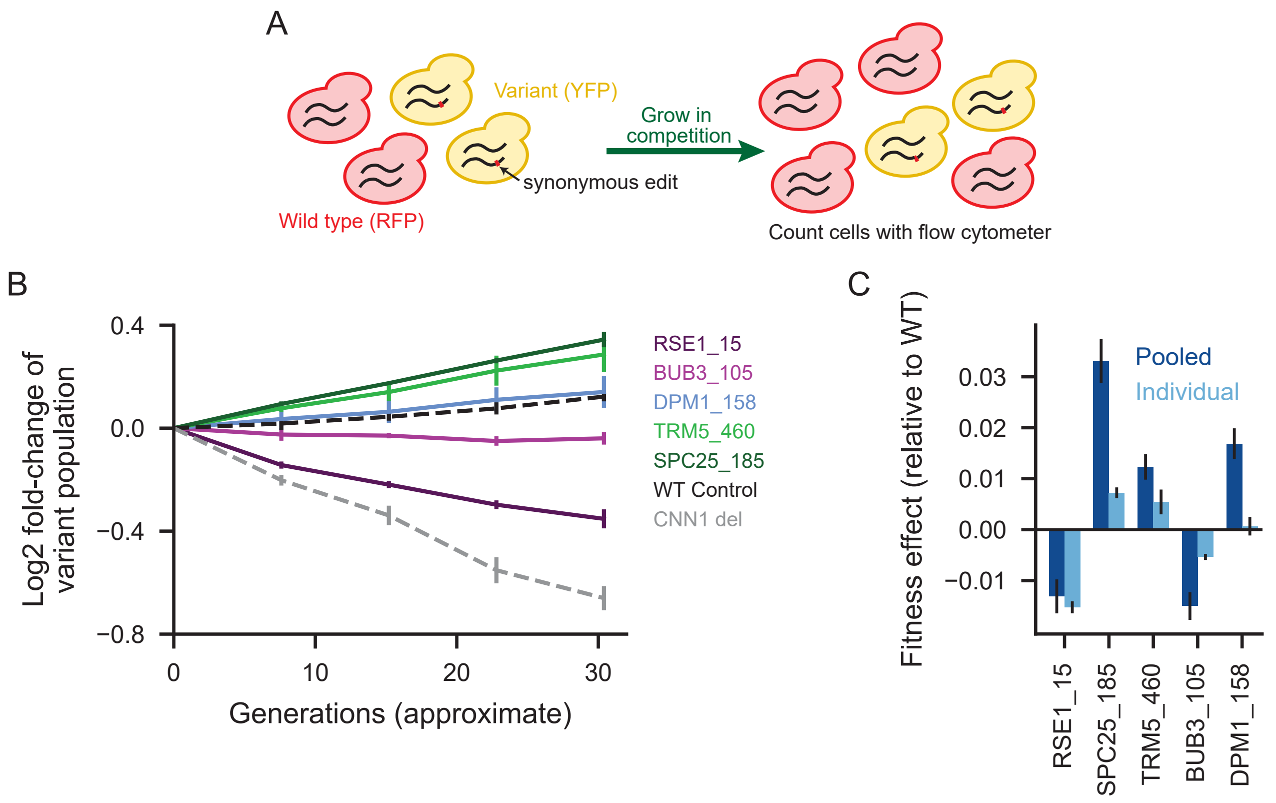

Figure S3: Paired fluorescently labeled competition. A) Yellow-fluorescent cells containing a specified synonymous slow-to-fast mutation were grown in competition with red-fluorescent wild-type cells. A flow cytometer was used to count the proportion of variant cells over time. B) The change in proportion of variant cells over thirty generations for five synonymous slow-to-fast mutations, a yellow-fluorescent wild-type control, and a gene deletion control known to have a weak growth defect. C) The measured fitness effects of the synonymous variants in the paired head-to-head competition compared to the fitness effects observed in a pooled competition.
